# Effect of Mahuang Gancao Ganjiang Decoction on Fusion and Fission of Mitochondria and Apoptosis of Lymphocytes in Mice under Cold Stress

**DOI:** 10.1155/2017/5132963

**Published:** 2017-01-16

**Authors:** Longyun Chen, Huimin Chen

**Affiliations:** Hubei University of Chinese Medicine, Basic Medical College, Wuhan 430065, China

## Abstract

Mahuang Gancao Ganjiang Decoction (MGGD) can effectively alleviate the symptoms of the patients suffering from exogenous cold stress. However, the curative mechanism has not been fully clarified. This study was designed to investigate the effect of MGGD on the apoptosis of lymphocytes induced by cold stress in mice. The model mice were randomly divided into four groups: the normal control group (no handling mice), cold stress group, MGGD + cold stress group, and MGGD group. Lymphocytes of the mice were isolated from the peripheral blood. Electron microscopy analysis revealed cold stress resulted in mitochondrial fragmentation. Accompanied with the change of morphology of mitochondria, ATP production and the activity of respiratory chain complex decreased in these cells. Western blot analysis showed that these cells expressed decreased fusion-related proteins Mitofusin 1 (Mfn1), Mitofusin 2 (Mfn2), and optic atrophy protein-1 (Opa-1) and increased fission-related proteins dynamin-related protein 1 (Drp1) and fission 1 (Fis-1); our results also show that decreased mitochondrial fusion induces cell apoptosis during cold stress. Meanwhile, we found MGGD can inhibit cell apoptosis induced by cold stress through regulating expression level of Mfn1, Mfn2, Drp1, Fis-1, and Opa-1. These findings are very significant for understanding how MGGD regulates cold-stress-induced cell apoptosis.

## 1. Introduction

The metabolism and growth of cells can be affected by cold stress significantly. In previous studies, it has been demonstrated that cold stress can induce apoptosis in cultured mammalian cells [1–3]. In the life and death of mammalian cells, mitochondria play very important roles [4, 5]. Because of the fusion and fission of mitochondria, the population and morphology of mitochondria are dynamic in mammalian cells. Imbalance of fusion and fission is always accompanied by mitochondrial dysfunction. The mitochondrial fusion and fission are controlled by the specific molecules. Mfn1, Mfn2, and Opa-1 are essential for mitochondrial fusion. Deletion of Mfn1 and Mfn2 leads to severe mitochondrial fragmentation and low levels of mitochondrial fusion [6, 7]. Opa-1 mediates the fusion of mitochondrial inner membrane; the decrease of Opa-1 expression can cause cell apoptosis [6]. The fission of mitochondria is regulated by Drp1 and Fis-1 [5, 8].

Currently, a series of clinical trials have shown that the representative formula Mahuang Gancao Ganjiang Decoction (MGGD) in Traditional Chinese Medicine (TCM) can significantly ease the symptoms and improve the quality of life in patients suffering from exogenous cold stress [9, 10]. However, the mechanism of the treatment effect has not been elucidated, which limits the application of MGGD on a larger scale.

Imbalance of mitochondrial fusion and fission was found in mammalian cells under cold stress, which is recognized to be related to cell apoptosis. Previous study revealed that overexpression of both fusion proteins Mfn1 and Mfn2 can delay apoptosis [11, 12]. The mechanism of apoptosis induced by the cold stress still needs to be studied. Further whether MGGD-formula can work on the apoptosis also needs to be explored. Hence, the aims of this study were to investigate the effect of MGGD on cold-stress-induced apoptosis and related mechanism.

## 2. Materials and Methods

### 2.1. Animals and Lymphocytes Preparation

SPF grade Kunming mice were purchased from the experimental animal center of Hubei University of traditional Chinese medicine. The medical laboratory animal certificate number is SCXK (Hubei) 2008-0005. The mice were randomly divided into 4 groups: (a) control group: the mice were kept under a 12-hour light/dark cycle (lights on at 8:00 a.m.) with room temperature (20–23°C) and humidity (50% ± 5%); (b) cold stress group: the mice were put under 4°C for 4 hours in artificial climate box with humidity (50% ± 5%) each day in a week; (c) MGGD + cold stress group: the mice were put under 4°C for 4 hours daily and then were conducted by intragastric administration of MGGD; (d) MGGD group: the mice were kept under room temperature and then were conducted by intragastric administration of MGGD. The capacity of intragastric administration was 1 ml/100 g body weight. In our study, female mice were excluded. All manipulations were performed between 7:00 a.m. and 12:00 a.m. every day to minimize the influence of circadian rhythms.

After treatment, all the mice in the four groups were injected with an appropriate amount of sodium for anaesthesia. One eye of anesthetic mice was pulled off by tweezers, and the fresh blood was collected by the 5 ml EP tube with anticoagulants. Lymphocytes separation was performed using a mouse T Cell Isolation Kit (Miltenyi) according to the manufacturer's instructions. After the separation, lymphocytes were used in the following experiments.

### 2.2. MGGD Composition and Administration

MGGD-formula was composed of the following traditional Chinese herbal medicines as recorded by the “Formulas of Chinese Medicine” [13]: Mahuang (Ephedra) 20 g, Gancao (Liquorice) 30 g, and Ganjiang (dried ginger) 30 g, which were composed in 2 : 3 : 3 proportion. All raw materials in MGGD-formula were prepared and examined according to quality control standard of Chinese Pharmacopoeia [14]. It was manufactured by the Preparation Room for TCM of the Chinese Medicine Hospital of Hubei Province. The mice in the MGGD-formula group were given intragastric administration with MGGD-formula (1 ml/100 g/d). The control group and cold stress group were given distilled water.

### 2.3. Assessment of Mitochondrial Morphology with Electron Microscopy

Lymphocytes were collected and fixed with 2.5% glutaraldehyde on ice for 2 h. Then cells were treated by 2% osmium tetroxide and dehydrated with sequential washes with 50%, 70%, 90%, 95%, and 100% ethanol. Cells were block mounted and thinly sliced. Cells of thin sections were examined under electron microscope.

### 2.4. Determination of Intracellular ATP Content

The lymphocytes were washed with cold PBS three times and 200 *μ*l of the lysis buffer was added. The cells were L centrifuged (12,000 ×g, 10 minutes, 4°C), and supernatants were harvested. 100 *μ*l ATP working solution was added to the supernatants in 1.5 ml tube, and the tube was incubated at room temperature for 5 minutes. The ATP content in samples was measured with Luminometer. At the same time, protein concentration was measured via BCA method. Then the ATP concentration is converted into the form of *μ*mol/g protein.

### 2.5. Mitochondrial Membrane Potential Detection (JC-1)

The lymphocytes of peripheral blood were washed by ice-cold PBS three times and centrifuged at 1500 rpm for 5 min. 5 × 10^5^ cells were suspended in 0.5 ml cell culture medium and dyed by 0.5 ml JC-1 dyeing fluid. Cells were incubated at 37°C for 20 minutes. After the incubation, cells were centrifuged at 1200 rpm for 3 min, and supernatants were discarded. Cells were washed by JC-1 dyeing buffer 2 times and suspended by 0.5 ml JC-1 dyeing buffer. The mitochondrial membrane potential of lymphocytes was detected by flow cytometry.

### 2.6. Measurement of Mitochondrial Respiratory Chain Complex Activity

Lymphocytes were homogenized with 1 ml of potassium phosphate buffer (100 mM, pH 7.4) at 4°C. The homogenates were centrifuged (1,500 ×g, 5 min, 4°C), and the supernatants were collected. The content of mitochondrial protein was measured via the bicinchoninic protein assay. All activities were determined on a spectrophotometer in potassium phosphate buffer. Complex I activity (NADH-CoQ reductase) was determined by measuring the disappearance of NADH at 340 nm in the presence of decylubiquinone [15]. Complex II activity (Succinate Dehydrogenase) was evaluated by measuring the reduction of dichloroindophenol (DCIP) at 600 nm [16].

### 2.7. Immunofluorescence Analysis

Cell suspension was fixed by 4% paraformaldehyde and coated on the slides, dry naturally. The cells were washed three times with PBS. Then cells were treated by 0.1% Triton X-100 for 20 min and blocked with 3% BSA for 30 min. Specific primary Abs for Mfn1, Mfn2, or Drp1 were added and incubated for 1 h at room temperature. Mito-Tracker Green was used to detect the mitochondria in lymphocytes. Cells were then washed with 1% BSA for three times, followed by incubation with goat anti-rabbit IgG fluorescent secondary antibody for 1 h. Nucleus was stained with DAPI (4′,6-diamidino-2-phenylindole). Cells were observed under the fluorescence microscope.

### 2.8. SDS-PAGE and WB

Lymphocytes were harvested and lysed with 100 *μ*l of lysis buffer (20 mM Tris-HCl [pH 8.0], 100 mM NaCl, 1.9% [wt/vol] Triton X-100, 1 mM 1,4-dithiothreitol, and 5% [vol/vol] glycerol) for 30 min on ice. After centrifugation for 30 min at 4°C, the supernatants were collected. The concentration of protein was determined via the Bradford method. Equal amounts of protein were separated by 10% SDS-PAGE and transferred onto a nitrocellulose membrane (GE Healthcare). Membrane was blocked with 5% nonfat milk and incubated with the primary antibodies, followed by HRP-conjugated goat anti-rabbit secondary antibodies.

### 2.9. Detection of Apoptosis

The lymphocytes of mice were washed by PBS three times and centrifuged at 1500 rpm, 5 min. Cells were suspended by binding buffer 500 *μ*l. Then the mixture containing 5 *μ*l AnnexinV-FITC and 5 *μ*l PI was added into cell suspension. Cells were incubated at room temperature for 5~15 min in dark. The number of apoptotic cells was determined by flow cytometry.

## 3. Results

### 3.1. Effect of MGGD on the Mitochondrial Morphology under Cold Stress

**Figure 1:**
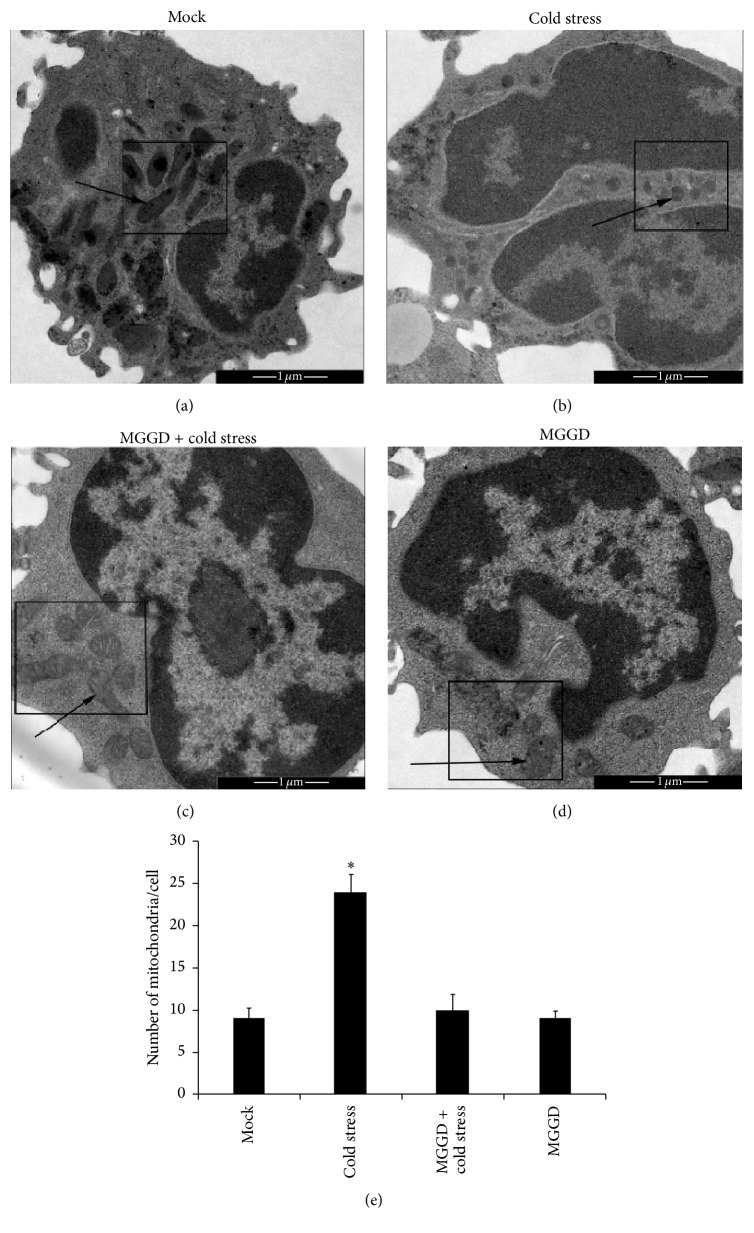
Morphology of mitochondria in lymphocytes. Lymphocytes were isolated from the peripheral blood of mice and analyzed for the morphology of mitochondria via electron microscopy. (a) Morphology of mitochondria in lymphocytes of mice which were kept under normal temperature (15–20°C). (b) Morphology of mitochondria in lymphocytes of mice which were kept under 4°C about 4 hours each day. (c) Mice suffering from cold stress were treated by MGGD; then the morphology of mitochondria in lymphocytes was observed under electron microscopy. (d) Mice were treated by MGGD alone; then the morphology of mitochondria in lymphocytes was observed. (e) The number of mitochondria/cell was counted. ^*∗*^*p* < 0.05 versus control group.

Previous study has demonstrated that cold stress can affect the normal morphology of mitochondria [17]. In our study, we observed the cold stress causes the mitochondrial fragmentation. Under cold stress, mitochondrial morphology became shorter and vacuole structure (Figures 1(a) and 1(b)). We also noticed that number of mitochondria/cell increased under cold stress (Figure 1(d)). These indicated that the fission of mitochondria increased because of the cold stress. After being treated by MGGD, the damage of mitochondria in the mice reduced during cold stress. In Figure 1(c), vacuolization of mitochondria was not observed obviously, and the cristae of mitochondria were visible in lymphocytes of the mice conducted by intragastric administration of MGGD. These results demonstrated that MGGD-formula can reduce the effect caused by cold stress on mitochondrial morphology.

### 3.2. Effects of MGGD on Cold Stress Induced Decreasing of ATP Content and Mitochondrial Membrane Potential

**Figure 2:**
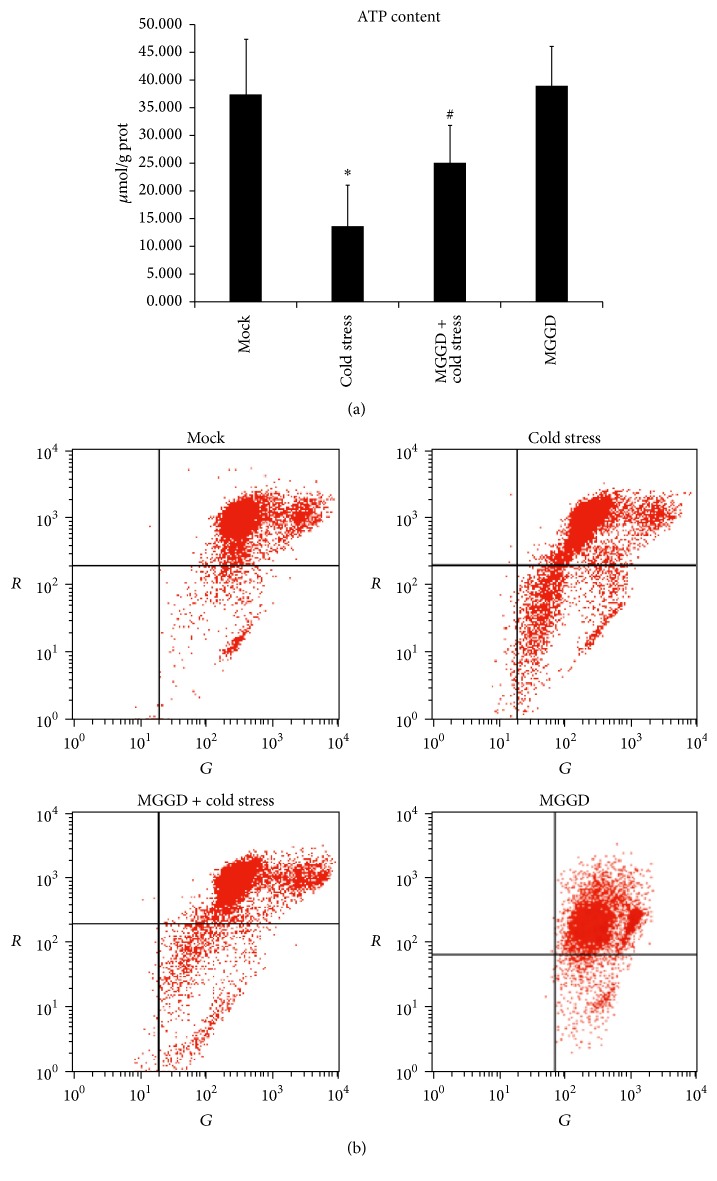
The ATP content and mitochondrial membrane potential of lymphocytes. (a) Cold stress caused the decrease of ATP content in lymphocytes. The ATP content of lymphocytes recovered after the mice treated by MGGD. ^*∗*^*p* < 0.05 versus control group; ^#^*p* < 0.05 versus cold stress group. (b) Mitochondrial membrane potential of lymphocytes was measured by flow cytometry. The values mentioned in the lower right corner of each flow cytometric dot-plot indicate how much membrane potential decreased. Representative dot-plots of three independent experiments are shown. Compared with the mice kept under normal temperature, the mice suffering from cold stress had low mitochondrial membrane potential in lymphocytes. MGGD can make the mitochondrial membrane potential of lymphocytes recover to a certain extent.

The change of mitochondrial morphology is closely related to its function. Mitochondrial membrane potential is an index to evaluate the function of mitochondria. According to previous study, when the mitochondrial fission increased, ATP synthesis and mitochondrial membrane potential will decrease [18–20]. In our experiment, both the ATP content and the mitochondrial membrane potential of lymphocytes decreased in cold stress group (Figures 2(a) and 2(b)). As shown in Figure 2(b), the mitochondrial membrane potential of cold stress group decreased about 20% compared to that of control and MGGD group. After the mice suffering from cold stress were treated by MGGD, the ATP content recovered. Meanwhile, the mitochondrial membrane potential increased about 10% compared to that of cold stress group. These data showed that MGGD can alleviate the effect caused by cold stress on ATP content and mitochondrial membrane potential.

### 3.3. MGGD Can Reduce the Effect of Cold Stress on Respiratory Chain Complex

**Figure 3:**
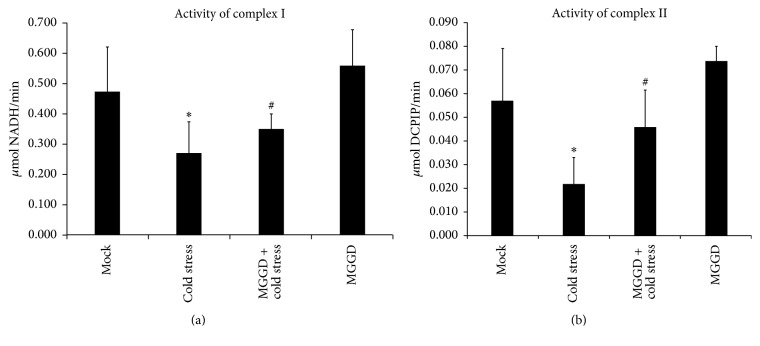
The activity of mitochondrial respiratory chain complex in lymphocytes. Cold stress had effect on the activity of mitochondrial respiratory chain complex. MGGD can reduce this kind of effect. (a) The activity of mitochondrial respiratory chain complex I. (b) The activity of the mitochondrial respiratory chain complex II. ^*∗*^*p* < 0.05 versus control group; ^#^*p* < 0.05 versus cold stress group.

The activity of respiratory chain complex is responsible for the mitochondrial membrane potential and ATP content in lymphocytes. Based on the mitochondrial respiratory chain complex activity detection, results showed that cold stress reduced the activity of complexes I and II by 42% and 61%, respectively (*p* < 0.05). After being treated by MGGD, the activity of complexes I and II recovered to 74% and 80%, compared with the control group (*p* < 0.05) (Figures 3(a) and 3(b)). These data showed that respiratory chain complex activity decreased because of the imbalance of mitochondrial fusion and fission induced by cold stress, and MGGD can reduce this kind of effect.

### 3.4. Effects of MGGD on the Expression of Mfn1, Mfn2, Drp1, Fis-1, and Opa-1

**Figure 4:**
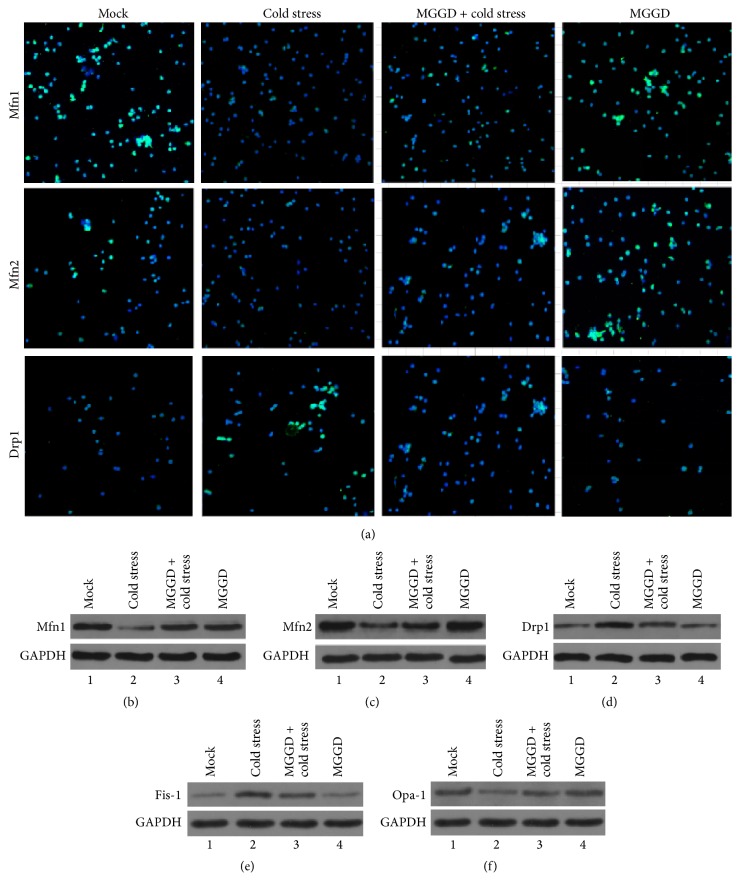
The expression level of Mfn1, Mfn2, Drp1, Fis-1, and Opa-1. (a) Mfn1, Mfn2, and Drp1 in lymphocytes were detected by specific antibody and labeled by FITC via immunofluorescence as described in Materials and Methods. The nucleus was stained with DAPI. (b, c, and d) The expression levels of Mfn1, Mfn2, and Drp1 in lymphocytes were analyzed via western blot. GAPDH was used as control. (e and f) Expression of Fis-1 and Opa-1 in lymphocytes was detected via western blotting with specific antibody.

Until now, the impairment and dysfunction of mitochondria had been demonstrated to be related to the imbalance of its fusion and fission. The fusion and fission of mitochondria are closely related to the Mfn1, Mfn2, Drp1, Fis-1, and Opa-1. In order to check whether the expression of Mfn1, Mfn2, and Drp1 was affected by cold stress or MGGD, the Mfn1, Mfn2, and Drp1 in lymphocytes were labeled by FITC and observed under the fluorescence microscope. From the density of the fluorescence, we can see that the expression level of Mfn1 and Mfn2 decreased in lymphocytes under cold stress, but the expression level of Drp1 increased. In MGGD-formula group, we observed increasing expression level of Mfn1 and Mfn2; meanwhile expression level of Drp1 decreased (Figure 4(a)). We also detected the expression level of Mfn1, Mfn2, and Drp1 by western blotting. The results of western blotting are consistent with immunofluorescence (Figures 4(b), 4(c), and 4(d)). Western blotting also revealed that cold stress caused the increased Fis-1 protein and decreased Opa-1 protein in lymphocytes (Figures 4(e) and 4(f)). These results suggested that MGGD affects the mitochondrial fusion and fission through regulating the expression level of Mfn1, Mfn2, Drp1, Fis-1, and Opa-1.

### 3.5. MGGD Has Effect on the Colocalization of Drp-1 and Mitochondria

**Figure 5:**
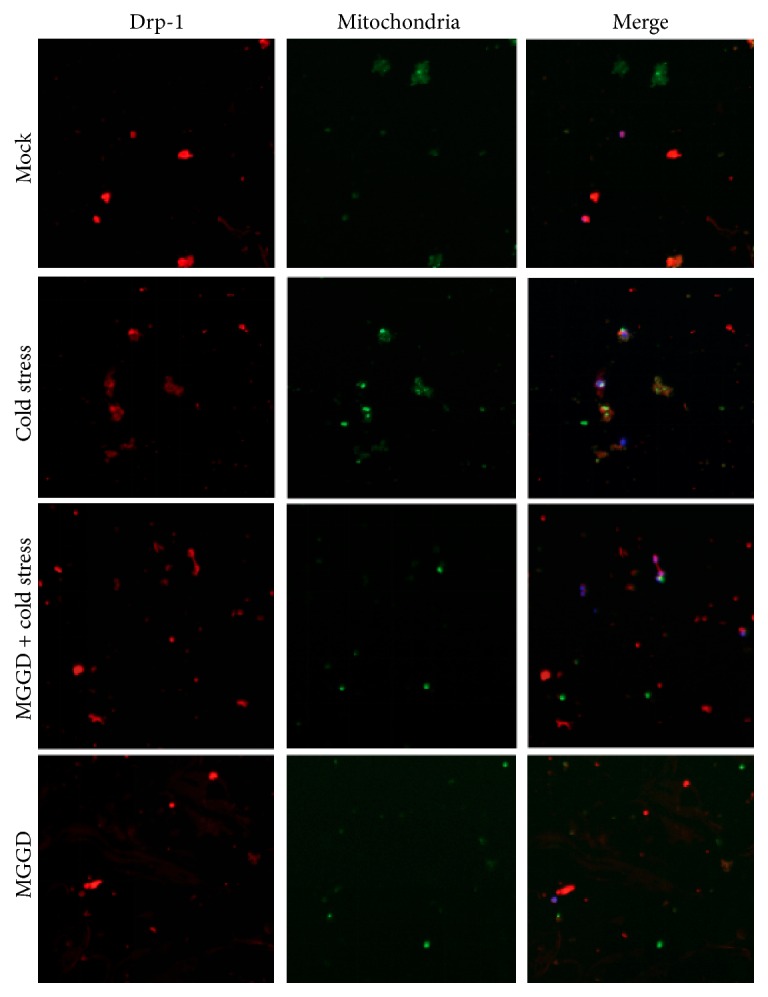
The colocalization of Drp-1 and mitochondria was analyzed. Mitochondria were labeled by Mito-Tracker Green. Drp1 in lymphocytes were detected by specific antibody and labeled by Cy3 as described in Materials and Methods.

During the fission of mitochondria, Drp1 was recruited by Fis-1 and translocated from cytoplasm to the mitochondrial outer membrane. Drp1 distributed at the potential division sites of mitochondrial outer membrane in the form of ring [8]. As shown in Figure 5, Drp-1 gathered around the mitochondria under the cold stress. However, Drp-1 mainly distributed in the cytoplasm in other three groups. This suggested that MGGD can reduce the fission of mitochondria through inhibiting the translocation of Drp-1.

### 3.6. MGGD Can Weaken the Cold-Stress-Induced Apoptosis

**Figure 6:**
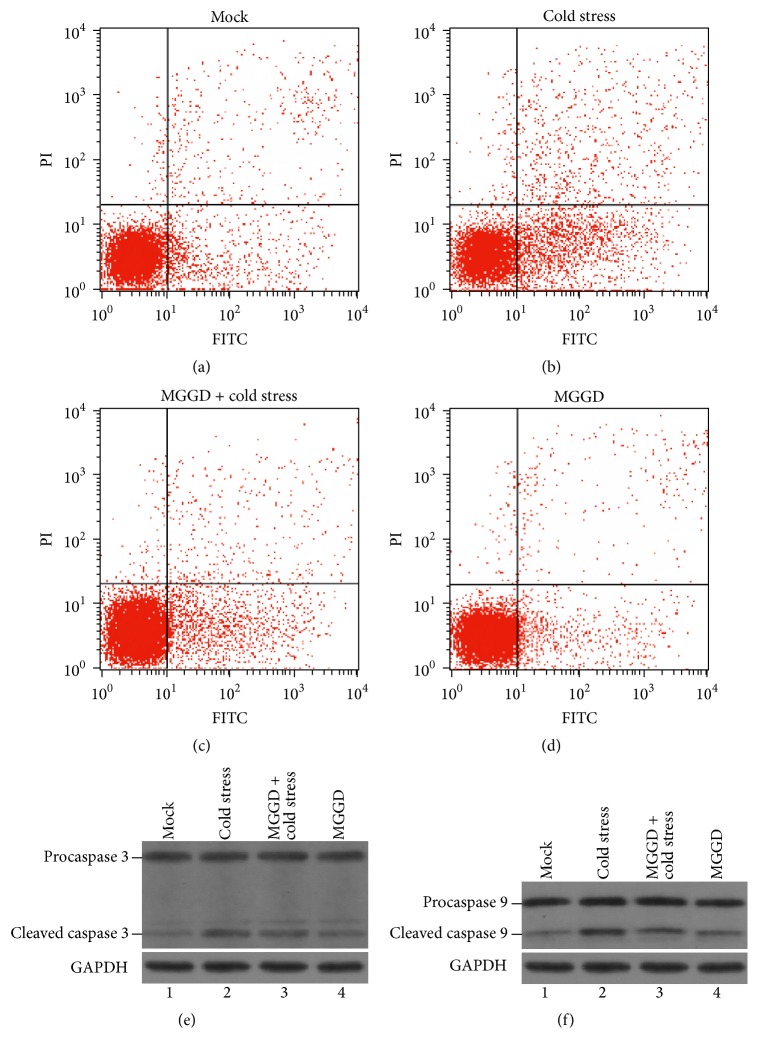
The apoptosis of lymphocytes was detected by flow cytometry. (a, b, c, and d) The values mentioned in the upper right corner and lower right corner of each flow cytometric dot-plot indicate the number apoptotic cells. Representative dot-plots of three independent experiments are shown. (e and f) The activation of caspases 3 and 9. Procaspase 3, procaspase 9, cleaved caspase 3, and cleaved caspase 9 protein levels were analyzed by western blotting.

The normal function of mitochondria is very important for the growth of cells. In our study, we found that cold stress affected the normal function of mitochondria, and MGGD can alleviate this kind of effect. So we want to check if MGGD can help the cells survive during cold stress. The lymphocytes in peripheral blood of four groups of mice were isolated, and the number of apoptotic cells was detected by flow cytometry. From Figures 6(a) and 6(b), we can see that the cold stress can induce the apoptosis of lymphocytes. The number of apoptotic lymphocytes decreased in MGGD-formula group (Figure 6(c)). The results of western blotting showed that activation of caspases 3 and 9 was induced by cold stress, and MGGD can reduce this effect (Figures 6(e) and 6(f)). These suggested that MGGD can alleviate the cold-stress-induced apoptosis.

## 4. Discussion

Cold cough is one of the most common clinical diseases. In China, Mahuang Gancao Ganjiang Decoction used in the treatment of typhoid symptoms has a long history. Our study indicated that MGGD can regulate the cold-stress-induced apoptosis. It has been demonstrated that the cold stress induces apoptosis in mammalian cells [1, 2, 21]. So the mechanisms of MGGD protecting cells from this type of injury in vivo need to be investigated. Our data presented here show that the response of lymphocytes to cold stress is associated with the imbalance of mitochondrial fission and fusion. MGGD can reduce this kind of effect through increasing the expression level of fusion-related protein and decreasing the expression level of fission-related protein.

MGGD as a popular formula in TCM was first recorded in Synopsis Prescriptions of Golden Chamber, which is a comprehensive medical book edited in Eastern Han Dynasty. Three Chinese herbs are included in the prescription, which are Mahuang (two Taels), Gancao (four Taels), and Ganjiang (two Taels). MGGD is a representative formula for treating asthma and anti-cold and recognized to be an alternative therapy for cold injury. Recently, a clinical study performed by Qin showed that MGGD could significantly relieve the discomfort in cold cough patients [10]. However, the curative mechanism has not been clearly demonstrated.

Mitochondria participate in many physiological processes. The responses of mammalian cells to cold stress have been demonstrated to be related to the changes in mitochondrial function [22]. Mitochondrial function is closely related to its morphology. Morphology of mitochondrial is in the dynamic balance of fusion and fission. In previous studies, it has been shown that disruption of fusion caused mitochondrial heterogeneity [6, 23, 24], and an increased rate of fission caused the mitochondrial fragmentation. In our study, we found that the mice response to cold stress resulted in the fragmented morphology of mitochondria in lymphocytes (Figures 1(a) and 1(b)). This indicated the increased rate of mitochondrial fission and the changes in its function. Mitochondrial dysfunction was always accompanied with the reduction of ATP content and its membrane potential [25]. So we decided to check these two parts in lymphocytes and found that both of them decreased under cold stress (Figures 2(a) and 2(b)). Our data also showed the activity of respiratory chain complexes I and II reduced under cold stress (Figures 3(a) and 3(b)). These findings demonstrated cold stress causes the disruption of normal function of mitochondria in lymphocytes. However our study also showed that these effects induced by cold stress were alleviated by MGGD-formula.

In order to find out the mechanism about how MGGD reduced the effect of cold stress on mitochondria, we detected the level of Mfn1, Mfn2, Drp1, Fis-1, and Opa-1 in lymphocytes of four groups of mice. In mammals, Mfn1, Mfn2, and Opa-1 are necessary for the mitochondrial fusion. OPA1 is required for the fusion of mitochondrial inner membrane. The loss of Opa-1 protein leads to dispersal of mitochondrial fragments and cell apoptosis. Once the Mfn1 and Mfn2 were deleted, it will lead to the reduction of fusion and fragmentation of mitochondria [6, 21]. Drp1 and Fis-1 are known as essential protein in mitochondrial fission. It is mainly in the form of poly and located in the cytoplasm [8, 26, 27]. According to the results of immunofluorescence and western blotting, the expression level of Mfn1, Mfn2, and Opa-1 reduced, and the expression of Drp1 and Fis-1 increased during cold stress. These are the reasons why the increase of number of mitochondria/cell was observed in lymphocytes of cold stress group. This phenomenon can be reduced by MGGD, because MGGD can increase the amount of Mfn1, Mfn2, and Opa-1 in cells. At the same time, the expression of Drp1 and Fis-1 was downregulated by MGGD (Figure 4).

It is well known that mitochondrion is an important executor of apoptosis; the abnormalities of its structure and function can lead to cell apoptosis. Previous study elucidated that the silencing of the Mfn2 can increase the Bcl-2 expression. Under normal condition, upregulated Bcl-2 could redeem the loss of Mfn2. However, Bcl-2 expression decreased in cells treated with Mfn2 siRNA could not redeem the loss of Mfn2 during cold stress, and the cell apoptosis occurred [28]. In our experiment, we also found cold stress can induce the apoptosis of lymphocytes (Figures 6(a) and 6(b)). As mitochondria are so important to cold-stress-induced apoptosis, maintaining their normal function is critical for protection of the lymphocytes against this process. Fortunately, MGGD was found to be able to protect lymphocytes against cold-stress-induced apoptosis at certain extent (Figure 6(c)).

Lymphocytes are very important for the immune response in mammals. The increasing of lymphocytes apoptosis may cause disorder of immune system and can not protect the organism from being invaded by germs or viruses effectively during cold exposure. Our findings indicated that MGGD may maintain the immune response through inhibiting the lymphocytes apoptosis in mice under cold stress.

In conclusion, the present study has demonstrated that MGGD was able to regulate the fusion and fission of mitochondria and the apoptosis of lymphocytes, mediated by regulating the expression level of Mfn1, Mfn2, and Drp1 under cold stress. Our results also suggested that this might be the mechanism of MGGD helping mice adapt to cold stress, at least in part.
